# Survival Analysis and its Associated Factors of Beta Thalassemia Major in Hamadan Province

**Published:** 2015-05

**Authors:** Reza Zamani, Salman Khazaei, Shahab Rezaeian

**Affiliations:** 1 Center for Disease Control and Prevention, Deputy of Health Services, Hamadan University of Medical Sciences, Hamadan, Iran;; 2 Department of Epidemiology and Biostatistics, School of Public Health, Tehran University of Medical Science, Tehran, Iran;; 3 Department of Epidemiology, School of Health, Shiraz University of Medical Sciences, Shiraz, Iran

**Keywords:** Survival, Thalassemia, Kaplan-Meier estimate, Iran

## Abstract

**Background:**

There currently is a lack of knowledge about the long-term survival of patients with beta thalassemia (BT), particularly in regions with low incidence of the disease. The aim of the present study was to determine the survival rate of the patients with BT major and the factors associated with the survival time.

**Methods:**

This retrospective cohort study was performed in Hamadan province, located in the west of Iran. The study included patients that referred to the provincial hospitals during 16 year period from 1997 to 2013. The follow up of each subject was calculated from the date of birth to the date of death. Demographic and clinical data were extracted from patients’ medical records using a checklist. Statistical analysis included the Kaplan-Meier method to analyze survivals, log-rank to compare curves between groups, and Cox regression for multivariate prognostic analysis.

**Results:**

A total of 133 patients with BT major were enrolled, 54.9% of whom were male and 66.2% were urban. The 10-, 20- and 30-year survival rate for all patients were 98.3%, 88.4% and 80.5%, respectively.

Based on hazard ratio (HR), we found that accompanied diseases (P=0.01), blood type (P=0.03) and residency status (P=0.01) were significant predictors for the survival time of patients.

**Conclusion:**

The survival rate of BT patients has improved. Future researches such as prospective designs are required for the estimation of survival rate and to find other prognostic factors, which have reliable sources of data.

## Introduction

Beta thalassemia (BT) major is one of the most common hereditary diseases in Iran. In our country, thalassemia is a momentous disease because of (i) being on the thalassemia world belt with the highest prevalence of thalassemia worldwide,^1^ (ii) inter-familial marriages (consanguinity),^2^ and (iii) short life of patients, involving a multidisciplinary therapeutic team and its socio-economic and health burden.

It has been estimated that 4.5% of the world populations are carriers of hemoglobinopathies.^3^ Middle Eastern countries, including Iran, are among the countries with a high concentration of thalassemia patients. The rate of the thalassemia carriers varies from 1% to 10% in different provinces of Iran with the national prevalence of 4.5%. It is estimated that every year, approximately 300 children with thalassemia major are born in Iran.^4^ The highest prevalence of BT has reported in cities around the Caspian Sea and Persian Gulf regions. The thalassemia programme was established in 1997 in Iran to create a general infrastructure for the prevention of genetic disorders.^5^

Based on the previous epidemiological studies, several factors have an effect on the survival rate of BT patients, such as gender, number of annual transfusions, level of hemoglobin, ferritin level, accompanied diseases, and ethnicity.^6-8^ On the other hand, a number of factors including poor compliance with deferoxamine, myocardial iron loading and heart failure contribute to the high mortality of BT major.^9-11^

As stated in literature, the treatment of BT patients has improved morbidity and mortality^12^^,^^13^ and the birth prevalence of BT major has also decreased during the past decades.^14^ However, the long-term survival remains poor, with data from southeast of Iran showing 40.7% of patients died by the age of 25 years.^7^ Consequently, the aim of the present study, which to the best of our knowledge is one of the first studies of its kind, was to determine the survival rate of patients with BT major and the factors associated with the survival time.

## Patients and Methods

A retrospective cohort study was performed in Hamadan province (west of Iran), including patients that referred to the provincial hospitals during 16 year period from 1997 to 2013. The study was approved by the local Human Subject Review Board of Hamadan University of Medical Sciences.

According to the importance and effectiveness of the results from the previous surveys of thalassaemia prevention programme in other countries,^15-17^ this programme was added to the Iranian primary healthcare programme in 1991 and the pilot project started in some districts followed by the whole country in 1997.^5^^,^^18^ After 13 years, the evaluation of the programme showed that it is far from complete and failed to detect structural haemoglobin variants such as haemoglobin S. In addition, screening projects for the risk of sickle cell disorders are now in progress as a pilot study.^5^ Hence, based on the census method, only the information of beta thalassemia major patients was investigated according to patients’ records. The follow up of each individual (in person-years) was calculated from the date of birth to the date of death. The non-native BT patients were considered as exclusion criteria for the survival analysis. The patients with unknown current situation, immigration, or those who had applied for bone marrow transplantation were considered censored observations.

Data were collected using a checklist. Demographic and clinical data such as age, sex, place of residence, consanguinity, diagnosis date, date of death (if applicable), blood group, type of RH, ferritin and hemoglobin level, blood transfusion, date of follow-up, kind of received blood (filtration, washed or unknown), and accompanied diseases were extracted from the medical records of patients.

Survival curves were calculated by the Kaplan-Meier method and survival differences were tested using the log-rank test. A Cox-proportional hazards model was used to compare survival rate of BT patients by independent predictors of disease-specific survival. All statistical analyses at 95% confidence level were performed using Stata 11 software (StataCorp, TX, USA). 

## Results

A total of 133 patients with BT major were enrolled, 54.9% of whom were male and 66.2% were urban. 36.1% of patients had blood group A and 92.5% were positive RH. Accompanied diseases such as diabetic, heart disease, and hepatitis C were observed in 26.3% of the patients. Table 1 summarizes the descriptive statistics of thalassemia patients.

**Table 1:** Descriptive statistics of thalassemia patients in Hamadan province, Iran

**Variable**	**Number (%)**	**No. of death**	**Median survival±SD**	** P value ^a^**
Gender				
Male	73 (55.0)	11	18.0±8.7	0.11
Female	60 (45.0)	5	20.4±10.9	
Residency				
Urban	88 (66.2)	8	22.6±10.4	0.01
Rural	45 (33.8)	8	14.4±7.2	
Consanguinity				
No relation	35 (26.3)	5	21.3±9.8	0.39
First cousin	45 (33.8)	7	18.0±9.2	
Second cousin	53 (39.9)	4	20.3±10.5	
Blood group				
A	46 (34.6)	5	22.2±9.8	0.54
B	40 (30.0)	5	15.9±10.4	
AB	9 (6.8)	0	14.5±11.0	
O	38 (28.6)	6	20.0±8.9	
Type of RH				
Positive	122 (91.7)	15	20.4±9.8	0.92
Negative	11 (8.3)	1	12.9±9.6	
Kind of blood				
Filtrated	83 (62.4)	5	19.6±9.9	0.02
Other	50 (37.6)	11	20.1±9.7	
Level of ferritin				
≤2500	105 (78.9)	12	21.3±10.2	0.40
>2500	28 (21.1)	4	17.7±8.5	
Level of hemoglobin				
≤9	100 (75.2)	10	20.8±10.3	0.05
>9	33 (24.8)	6	17.3±8.0	
Accompanied diseases				
None	98 (73.7)	2	18.2±10.7	0.01
Diabetic	11 (8.2)	4	22.6±7.5	
Heart disease	11 (8.2)	4	20.0±6.9	
Hepatitis C	9 (6.8)	3	22.0±5.8	
Heart disease & Hepatitis C	3 (2.3)	2	19.7±11.9	
Diabetics & hepatitis C	1 (0.8)	1	22.8	

Data are shown as n (%) and median of survival±standard deviation (SD)

**Figure 1:**
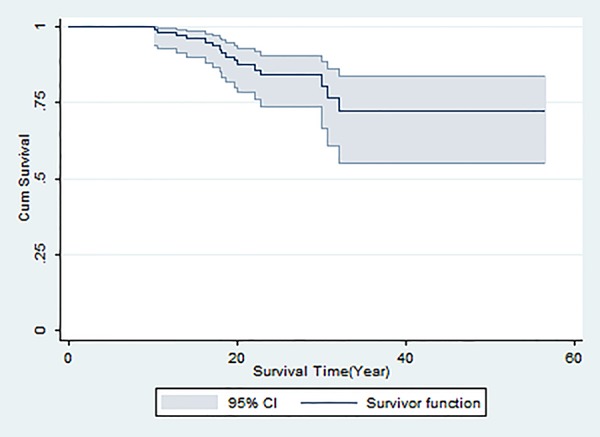
Overall Kaplan-Meier survival curve with 95% CI for thalassemic patients, Hamadan province, Iran.

The mean age of patients was 20.1 years (21.7 years in females and 18.7 years in males, P=0.08) ranged from 0.97 to 56.6 years. Of all patients, 16 deaths occurred and 30 individuals were censored during 356 to 20,676 days of the follow-up period. Figure 1 illustrates the Kaplan-Meier observed curve with 95% confidence interval for all BT patients in the study. The maximum survival was 56.6 years in females and 39.0 years in males. The 10-, 20- and 30-year survival rate for all patients were 98.3%, 88.4% and 80.5%, respectively.

As shown in table 1, there was no significant difference of survival years between different genders, blood groups, levels of ferritin and hemoglobin, consanguinity and the positive and negative blood RH. However, the survival time of the patients showed a significant relationship with the residency status (median survival in rural 14.4 vs. 22.6 in urban, P=0.01), kind of received blood (P=0.02) and accompanied diseases (P=0.01).

The results of the univariate and multivariable analysis, using Cox’s proportional hazard model, including the seven factors (sex, residency, consanguinity, levels of ferritin and hemoglobin, kind of received blood and accompanied diseases) are shown in table 2. Based on both univariate and multivariable analyses, the results show that, rural residency and accompanied diseases are associated with statistically significant increased risk of death among BT patients.

**Table 2:** The result of univariate and multivariate Cox proportional hazards survival analysis in thalassemia patients

**Variable**	**Crude**	**P value**	** Adjusted ^a^**	**P value**
	**HR (95% CI)**		**HR (95% CI)**	
Gender				
Male	1.00	0.12	1.00	0.06
Female	0.43 (0.15, 1.25)		0.32 (0.10, 1.05)	
Residency				
Urban	1.00	0.01	1.00	0.04
Rural	4.56 (1.65, 12.60)		2.91 (1.02, 8.27)	
Consanguinity				
No relation	1.00	-	-	-
First cousin	0.70 (0.22, 2.20)	0.52	-	-
Second cousin	0.44 (0.13, 1.50)	0.18	-	-
Kind of blood			-	-
Filtrated	1.00	0.03	1.00	0.08
Other	3.11 (1.10, 9.97)		2.73 (1.10, 12.24)	
Level of ferritin				
≤2500	1.00	0.41	-	-
>2500	1.61 (0.52, 5.01)		-	
Level of hemoglobin				
≤9	1.00	0.06	1.00	0.05
>9	2.60 (0.93, 7.17)		3.10 (0.96, 9.96)	
Accompanied diseases				
No	1.00	0.01	1.00	0.01
Yes	14.58 (3.31, 64.22)		15.45 (3.36, 71.02)	

^a^Adjusted for all other variables in the table except for consanguinity & level of ferritin; HR: Hazard ratio; CI: Confidence interval

**Figure 2:**
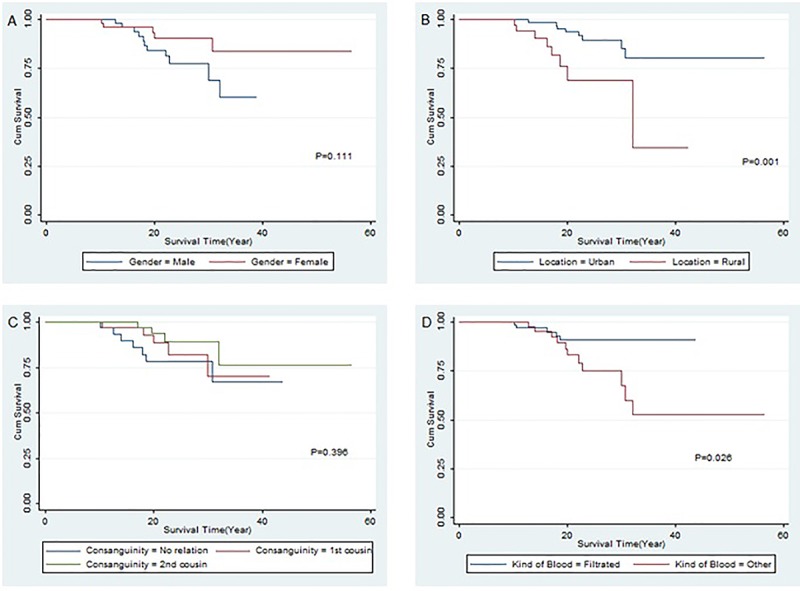
Kaplan-Meier survival curves of thalassemic patients. A: By gender; B: By region; C: By consanguinity status; D: By kind of transfused blood.

The kind of received blood as one of the prognostic factors of the long-term survival was also shown in unadjusted hazards ratio model. Kaplan-Meier survival curves for thalassemic patients by gender, region, consanguinity status, and kind of blood transfusion are shown in figure 2.

## Discussion

In the present study, we have investigated the survival times of the 133 patients with BT major and evaluated the relationship between the survival time and the relevant factors. The median survival time is 20.0 years (range: 0.97-56.6 year) and 12.0% BT cases died within the study period. In addition, the 10-, 20-, and 30-year survival rates of the surveyed BT were 98.3%, 88.4% and 80.5%, respectively. We report an excellent survival rates for individuals with thalassemia, compared to a similar study of 578 BT patients in southeast of Iran, that shows the median survival time of 20- and 25-year of survival rates are 81.2% and 59.3%, respectively.^7^

No statistical difference in the survival time is observed between the genders, though the median survival time of female BT patients is longer than that of males (median survival in female 20.4 vs. 18.0 in male, P=0.11). Although, in other studies,^7^ male BT cases seem to have a relatively longer survival than females (maximum survival of 43 years in male vs. 25 years in female), there is also no statistical difference. In contrast, another study,^6^ evaluated the interactions between gender, birth cohort, complications and ferritin on survival, revealed that females have a better survival than males (survival at the age of 25 years 92.2% vs. 83.5%, P=0.01).

Based on the results from previous studies, serum ferritin level (persistently above 2500 ng/mL) is associated with the reduced survival in thalassemia.^6^^,^^7^ Borgna-Pignatti et al.^6^ concluded that, the lower levels of ferritin are predictive of a better prognosis in patients with BT major. In our study, in spite of a higher proportion of ferritin level less than 2500 ng/mL (78.9%), there was no significant difference between ferritin level and survival time. We also showed that there is no difference in mean serum ferritin levels between males and females. Other researchers have reported a similar result, which suggests that iron stores did not differ significantly between the sexes.^19^

The relatively poor survival of BT patients is probably due to a complex combination of living with a chronic disease. This result was seen in previous studies.^7^^,^^20^^,^^21^ Borgna-Pignatti et al.^22^ reported that the prevalence of complications in Italian patients includes heart failure in 7%, hypogonadism in 55%, hypothyroidism in 11% and diabetes in 6%. In addition, Chern et al.^21^ indicated that among the living BT patients over the age of 15, hypogonadotropic hypogonadism, HCV infection, diabetes, heart failure, and arrhythmia are the common complications. The relation between accompanied diseases and the quality of life in patients with thalassemia has also been highlighted by other authors.^13^

Although, there are several risks associated with chronic blood transfusions such as the risk of transmitting infections, blood transfusion remains the cornerstone of treatment for patients with BT major.^19^ In contrast to other studies,^7^ the rate of receiving filtrated blood is lower in our population. Only 62.4% of our patients received filtrated blood and there was a significant relation between the survival time and the kind of transfused blood. This finding was lower than those reported by Roudbari et al.^7^ who reported a rate of receiving filtrated blood at 98.3% among transfusion-dependent thalassemia patients. They have also shown that the survival time is significantly different between the two kinds of transfused blood, such that those receiving filtrated blood survived longer than those who used washed blood. In another study only 32.2% of patients received filtrated blood.^9^

It was noticed that in approximately 73.7% of cases, the patients were the result of first- or second-cousin marriages. However, we observed no significant association between the survival time of beta-thalassemia and consanguinity. In Asady-Pooya et al.^23^ study to determine the demography of beta-thalassemia major in Shiraz (south of Iran), 49.5% of beta-thalassemic patients were the result of first- or second-cousin marriages. In addition, another study reported the rate of 67.4% consanguinity among Balouch population in the southeast of Iran.^24^ This may be explained by several reasons, mainly, the lack of knowledge about the increased risk of beta-thalassemia after familial marriages among population especially among young individuals. To improve the situation, public awareness on thalassemia is of great importance and should be taught in periodic sessions.

Interestingly, the results of the present study showed that the likelihood of long-term survival was significantly lower for rural patients (median survival in rural 14.4 years vs. 22.6 years in urban, P=0.01). There may be several probabilities that help to account for the difference in survival time. There is a disparity between rural and urban settings in terms of the availability of primary care services. There are only rural health centers/houses in rural areas and there is a differential access to healthcare among rural residents.

We retrospectively investigated the survival time of BT major patients between 1997 and 2013, which is the main strength of this study. However, due to low prevalence in Hamadan, a limited number of patients were available for this study. Hence, there was not sufficiently high statistical power to detect statistical significance. This could be considered as one of the limitations of the present study. 

## Conclusion

The results of this study show that mortality rates are decreasing in patients with thalassemia major. In other words, the survival rate of patients has improved. Despite the survival rate of BT patients is affected by several prognostic factors, but in our study, accompanied diseases, kind of transfused blood, and rural residency had a statistically significant association with survival time of BT patients. Finally, prospective designs are required for the estimation of survival rate and to find other prognostic factors, which have reliable sources of data. 

## References

[1] Abolghasemi H, Amid A, Zeinali S, Radfar MH, Eshghi P, Rahiminejad MS (2007). Thalassemia in Iran: epidemiology, prevention, and management. J Pediatr Hematol Oncol.

[2] Mokhtari R, Bagga A (2003). Consanguinity, genetic disorders and malformations in the Iranian population. Acta Biologica Szegediensis.

[3] Weatherall DJ, Clegg JB (2001). Inherited haemoglobin disorders: an increasing global health problem. Bull World Health Organ.

[4] Dehshal MH, Ahmadvand A, Darestani SY, Manshadi M, Abolghasemi H (2013). Secular trends in the national and provincial births of new thalassemia cases in Iran from 2001 to 2006. Hemoglobin.

[5] Samavat A, Modell B (2004). Iranian national thalassaemia screening programme. BMJ.

[6] Borgna-Pignatti C, Rugolotto S, De Stefano P, Zhao H, Cappellini MD, Del Vecchio GC (2004). Survival and complications in patients with thalassemia major treated with transfusion and deferoxamine. Haematologica.

[7] Roudbari M, Soltani-Rad M, Roudbari S (2008). The survival analysis of beta thalassemia major patients in South East of Iran. Saudi Med J.

[8] Ansar MM, Kooloobandi A (2002). Prevalence of hepatitis C virus infection in thalassemia and haemodialysis patients in north Iran-Rasht. J Viral Hepat.

[9] Bejaoui M, Guirat N (2013). Beta thalassemia major in a developing country: epidemiological, clinical and evolutionary aspects. Mediterr J Hematol Infect Dis.

[10] Tanner MA, Galanello R, Dessi C, Westwood MA, Smith GC, Nair SV (2006). Myocardial iron loading in patients with thalassemia major on deferoxamine chelation. J Cardiovasc Magn Reson.

[11] Modell B, Khan M, Darlison M (2000). Survival in beta-thalassaemia major in the UK: data from the UK Thalassaemia Register. Lancet.

[12] Modell B, Khan M, Darlison M, Westwood MA, Ingram D, Pennell DJ (2008). Improved survival of thalassaemia major in the UK and relation to T2* cardiovascular magnetic resonance. J Cardiovasc Magn Reson.

[13] Gollo G, Savioli G, Balocco M, Venturino C, Boeri E, Costantini M (2013). Changes in the quality of life of people with thalassemia major between 2001 and 2009. Patient Prefer Adherence.

[14] Haghpanah S, Nasirabadi S, Rahimi N, Faramarzi H, Karimi M (2012). Sociocultural challenges of beta-thalassaemia major birth in carriers of beta-thalassaemia in Iran. J Med Screen.

[15] Maggio A, Caronia F, Orlandi F (1992). Prenatal diagnosis of haemoglobinopathies in Sicily. Lancet.

[16] Angastiniotis MA, Hadjiminas MG (1981). Prevention of thalassaemia in Cyprus. Lancet.

[17] Modell B, Ward RH, Fairweather DV (1980). Effect of introducing antenatal diagnosis on reproductive behaviour of families at risk for thalassaemia major. Br Med J.

[18] Moradi G, Ghaderi E (2013). Chronic disease program in Iran: Thalassemia control program. Chron Dis J.

[19] Telfer P, Coen PG, Christou S, Hadjigavriel M, Kolnakou A, Pangalou E (2006). Survival of medically treated thalassemia patients in Cyprus. Trends and risk factors over the period 1980-2004. Haematologica.

[20] Cunningham MJ, Macklin EA, Neufeld EJ, Cohen AR, Thalassemia Clinical Research Network (2004). Complications of beta-thalassemia major in North America. Blood.

[21] Chern JP, Su S, Lin KH, Chang SH, Lu MY, Jou ST (2007). Survival, mortality, and complications in patients with beta-thalassemia major in northern Taiwan. Pediatr Blood Cancer.

[22] Borgna-Pignatti C, Cappellini MD, De Stefano P, Del Vecchio GC, Forni GL, Gamberini MR (2005). Survival and complications in thalassemia. Ann N Y Acad Sci.

[23] Asadi-Pooya AA, Doroudchi M (2004). Thalassemia major and consanguinity in Shiraz city, Iran. Turk J Haematol.

[24] Miri-Moghaddam E, Zadeh-Vakili A, Rouhani Z, Naderi M, Eshghi P, Khazaei Feizabad A (2011). Molecular basis and prenatal diagnosis of β-thalassemia among Balouch population in Iran. Prenat Diagn.

